# Segmental strain for scar detection in acute myocardial infarcts and in follow-up exams using non-contrast CMR cine sequences

**DOI:** 10.1186/s12872-022-02664-z

**Published:** 2022-05-18

**Authors:** Malgorzata Polacin, Mihaly Karolyi, Matthias Eberhard, Ioannis Matziris, Hatem Alkadhi, Sebastian Kozerke, Robert Manka

**Affiliations:** 1 Institute of Diagnostic and Interventional Radiology, University Hospital Zurich, University of Zurich, Raemistrasse 100, 8091 Zurich, Switzerland; 2 Institute for Biomedical Engineering, University and ETH Zurich, Gloriastrasse 35, 8092 Zurich, Switzerland; 3 Department of Cardiology, University Heart Center, University Hospital Zurich, University of Zurich, Raemistrasse 100, 8091 Zurich, Switzerland

**Keywords:** Cardiac magnetic resonance, Acute myocardial infarction, Ischemic heart disease, Feature tracking

## Abstract

**Background:**

The purpose of the study was to investigate feasibility of infarct detection in segmental strain derived from non-contrast cardiac magnetic resonance (CMR) cine sequences in patients with acute myocardial infarction (AMI) and in follow-up (FU) exams.

**Methods:**

57 patients with AMI (mean age 61 ± 12 years, CMR 2.8 ± 2 days after infarction) were retrospectively included, FU exams were available in 32 patients (35 ± 14 days after first CMR). 43 patients with normal CMR (54 ± 11 years) served as controls. Dedicated software (Segment CMR, Medviso) was used to calculate global and segmental strain derived from cine sequences. Cine short axis stacks and segmental circumferential strain calculations of every patient and control were presented to two blinded readers in random order, who were advised to identify potentially infarcted segments, blinded to LGE and clinical information.

**Results:**

Impaired global strain was measured in AMI patients compared to controls (global peak circumferential strain [GPCS] *p* = 0.01; global peak longitudinal strain [GPLS] *p* = 0.04; global peak radial strain [GPRS] *p* = 0.01). In both imaging time points, mean segmental peak circumferential strain [SPCS] was impaired in infarcted tissue compared to remote segments (AMI: *p* = 0.03, FU: *p* = 0.02). SPCS values in infarcted segments were similar between AMI and FU (*p* = 0.8). In SPCS calculations, 141 from 189 acutely infarcted segments were accurately detected (74.6%), visual evaluation of correlating cine images detected 43.4% infarcts. In FU, 80% infarcted segments (91/114 segments) were detected in SPCS and 51.8% by visual evaluation of correlating short axis cine images (*p* = 0.01).

**Conclusion:**

Segmental circumferential strain derived from routinely acquired native cine sequences detects nearly 75% of acute infarcts and 80% of infarcts in subacute follow-up CMR, significantly more than visual evaluation of correlating cine images alone. Acute infarcts may display only subtle impairment of wall motion and no obvious wall thinning, thus SPCS calculation might be helpful for scar detection in patients with acute infarcts, when LGE images are not available.

## Background

Upon myocardial infarction, scar tissue is best visualized by cardiac magnetic resonance imaging (CMR) with late gadolinium enhancement (LGE) [1]. Intravenous application of gadolinium-based contrast agents is mandatory before acquiring LGE sequences. However, gadolinium should be used carefully in some patient groups, such as patients with severely reduced kidney function. Gadolinium-free options for the detection of ischemic myocardial scars are limited. One promising alternative is scar detection using regional myocardial deformation parameters [2, 3]. Myocardial deformation during cardiac contraction can be quantified by myocardial feature tracking (FT) based on routinely acquired, non-contrast cine sequences [4, 5]. Necrosis of myocytes after myocardial infarction with subsequent scar replacement disturbs mechanical properties of the myocardium with consecutively altered global and segmental strain [6]. Especially chronic myocardial scars with wall thinning and noticeable wall motion abnormality result in significant segmental strain impairment, which can be used to distinguish scar tissue from remote myocardium [2, 3, 7]. In contrast, acute infarcts might lack significant myocardial wall thinning and display less wall motion abnormalities in cine images. Therefore, the impact of acute and subacute infarcts on segmental strain needs to be further analyzed. In this study, global and segmental strain derived from non-contrast cine images was analyzed in patients with acute myocardial infarction (AMI) and in subacute follow-up (FU) exams and the practicability of using segmental strain for scar detection in both exams was investigated.

## Methods

### Study population

From July 2019 until December 2020 57 patients (15 female, mean age 61 ± 12 years) with AMI in CMR (imaging 2.8 ± 2 days [range 0–6 days] after reperfusion therapy) were retrospectively assessed. In those patients, CMR was performed to evaluate extent of infarction after revascularization [8]. Thirty-two out of 57 patients had a FU exam (35 ± 14 days, [range 20–86 days]). Patients with concomitant primary cardiomyopathies (n = 2) or non-diagnostic LGE images (n = 3) were not enrolled. Fourty-three individuals (13 female, mean age 54 ± 11 years) with normal CMR examinations during the same time period were also retrospectively included. CMR referrals in the control group were exclusion of structural heart disease (n = 16) and exclusion of coronary artery disease (n = 27). Demographic characteristics of patients and controls are shown in Table 1a, b, respectively.

**Table 1:** Demographic characteristics. a: Patients versus controls and b: AMI versus follow-up

	AMI n = 57	Controls n = 43	*p* values
*(a)*			
Demographics			
Sex (male/female)	42/15	30/13	–
Age (years)	61 ± 12	54 ± 11	0.2
BSA (m^2^)	1.9 ± 0.4	1.8 ± 0.3	0.5
BMI	27 ± 5	25 ± 3	0.02
LV morphology			
LV-EDV (ml)	191 ± 23	171 ± 31	< 0.01
LV-ESV (ml)	81 ± 32	66 ± 21	< 0.01
LV-SV (ml)	83 ± 15	91 ± 15	0.02
LV-EF (%)	50 ± 8	59 ± 6	< 0.01
LV mass (g)	60 ± 14	51 ± 10	0.04
Global strain			
GPCS (%)	− 10.3 ± 3	− 19.9 ± 2	0.01
GPLS (%)	− 10.7 ± 5	− 18.9 ± 4	0.04
GPRS (%)	27.9 ± 5	39.8 ± 6	0.01
Affected coronary vessel			
RIVA			
Reperfused acute occlusion	28		
Reperfused acute-on-chronic stenosis	1		
Failed reperfusion	2		
Acute coronary dissection	2		
RCA—reperfused acute occlusion	13		
RCA—acute dissection	3		
LCX—reperfused acute occlusion	6		
LCX—failed reperfusion	2		
Myocardial infarcts			
Infarcted segments	189/896		
Viable (< 50% wall width infarcted)	3		
Non-viable (> 50% wall width infarcted)	186		
Scar burden (%)	23.4 ± 6		
Segments with myocardial edema only	27		

	AMI CMR	Follow-up CMR	*p* values
*(b)*			
n	32		
Sex (male/female)	23/9		
Age (years)	52 ± 7		
BSA (m^2^)	1.9 ± 0.5		
BMI	27 ± 4		
LV morphology			
LV-EDV (ml)	172 ± 19	184 ± 27	0.2
LV-ESV (ml)	80 ± 29	86 ± 26	0.8
LV-SV (ml)	89 ± 18	72 ± 18	0.5
LV-EF (%)	47 ± 10	51 ± 8	0.2
LV mass (g)	60 ± 10	53 ± 8	0.6
Global strain			
GPCS (%)	− 10.6 ± 2	− 9.5 ± 3	0.7
GPLS (%)	− 10.2 ± 5	− 10.9 ± 5	0.8
GPRS (%)	26.8 ± 6	29.8 ± 4	0.2
Myocardial infarcts			
Infarcted segments	118/512	118/512	
Viable (< 50% wall width infarcted)	1	16	0.02
Non-viable (> 50% wall width infarcted)	117	102	0.5
Scar burden (%)	25.1 ± 5	20.7 ± 4	0.6
Segments with myocardial edema only	10	–	–
Patients with infarcts detected in SPCS^a^	32/32	31/32	-

*BSA* body surface area, *BMI* body mass index, *LVEDV* left ventricular end-diastolic volume, *LVESV* left ventricular end-systolic volume, *LVSV* left ventricular stroke volume, *LVEF* left ventricular ejection fraction, *GPCS/GPLS/GPRS* global circumferential/longitudinal/radial strain

^a^Patients with infarcts in LGE served as gold standard

### CMR data acquisition

CMR exams were acquired on a 1.5 T MR (Achieva, Philips Healthcare). Cine balanced steady-state free precession (SSFP) images in long-axis geometries (2-, 3- and 4-chamber view) and in short axis orientation covering the entire left ventricle (LV) (field of view: 350 × 350 mm^2^; matrix: 300 × 300; repetition time/echo time: 3.0/1.5 ms; in-plane resolution 1.2 × 1.2 mm^2^; number of cardiac phases: 50; section thickness: 8 mm) were acquired for functional assessment of the LV. Edema-sensitive black-blood T2-weighted images with fat saturation in five short axis slices were acquired for visualization of myocardial edema [9]. Fifteen minutes after administration of gadolinium (0.2 mmol gadobutrol [Gadovist; Bayer Schering Pharma, Zurich, Switzerland] per kilogram body weight), LGE (inversion recovery gradient-echo sequence; field of view: 350 × 350 mm^2^; matrix: 234 × 234; repetition time/echo time: 7.4/4.4 ms; inversion time: 205–255 ms; flip angle: 20°; in-plane resolution: 1.5 × 1.5 mm^2^; section thickness: 8 mm) was performed in short axis and in 2-,3- and 4 chamber view.

### CMR data analysis

#### Strain analysis

Dedicated software (Segment v3.0 R7946, Medviso, Lund, Sweden) was used to calculate global and segmental strain derived from native cine sequences as previously described [3]. Duration of data loading, image registration, contouring of myocardial borders and strain calculation was 9 min 58 s ± 35 s (range 9 min 4 s–11 min 31 s) per patient or control, respectively. Blinded to patient information (patient or control) and to LGE images, all strain analyses were performed by one reader (reader A: 5 years of experience in cardiac imaging). Interobserver agreement was performed on 28 random cases by a second reader due to the semi-automatic nature of strain analyses (reader B: 2 years of experience in cardiac imaging, blinded to the results of reader A).

#### Infarct detection in circumferential strain calculations and in cine images

**Fig. 1:**
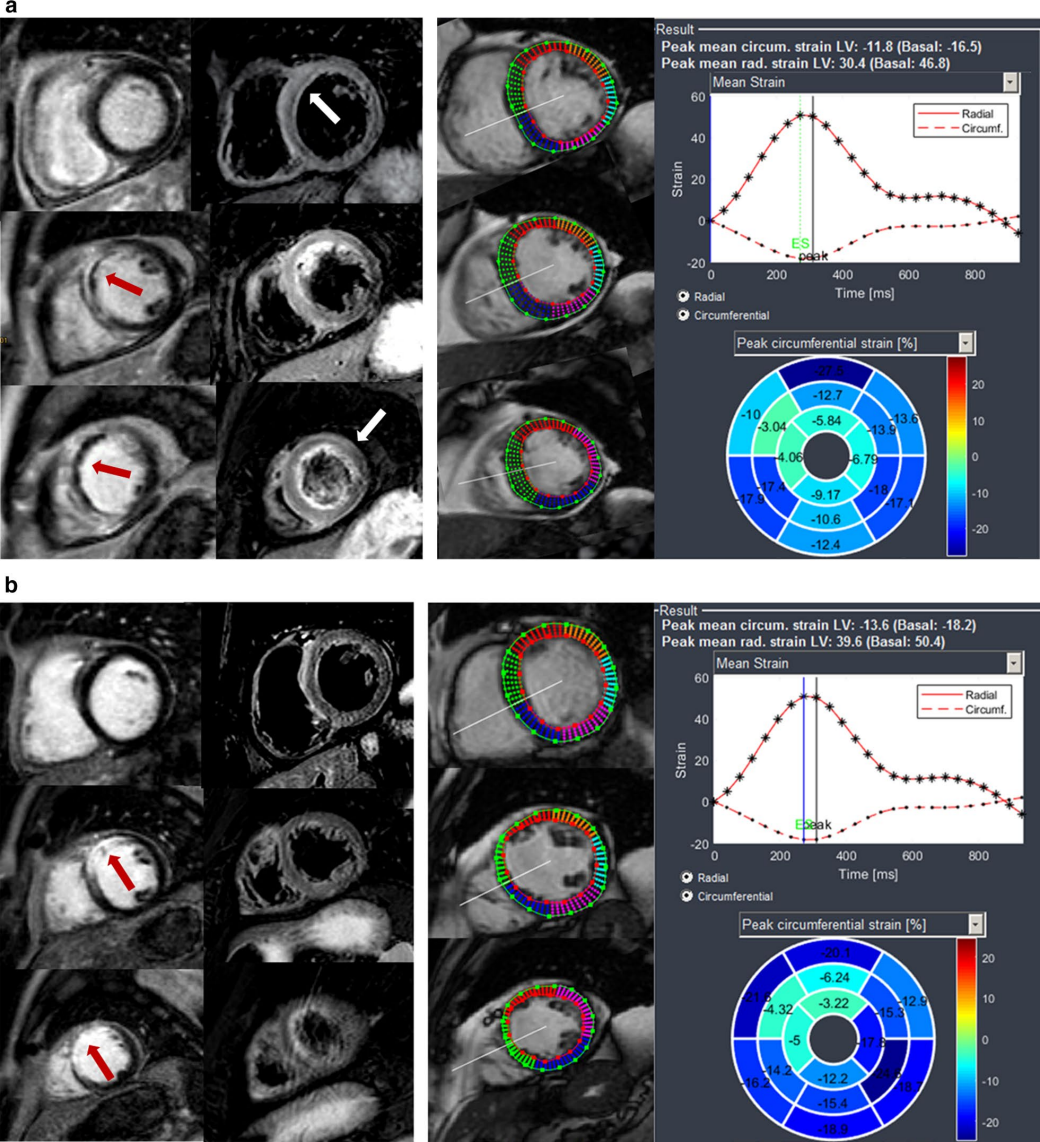
**a** 48-year old patient 2 days after infarction of the anteroseptal wall. Left column: LGE in segment 8, 13, 14 (red arrows), concomitant edema extends additionally into segments 2, 7, 16 (white arrows). Right column: Endo- and epicardially contoured basal, midventricular and apical cine short axis slices prepared for circumferential strain calculations with polar plot strain map. Infarcted segments display reduced SPCS values in the strain map. **b** 48-year old patient 35 days after infarction of the anteroseptal wall. Same patients as in **a**. Left column: LGE in segment 8, 13, 14 (red arrows), no concomitant edema; right column: Endo- and epicardially contoured basal, midventricular and apical cine short axis slices prepared for circumferential strain calculations with polar plot strain map. Infarcted segments display reduced SPCS values in the strain map

Reader A and B were advised to identify possibly infarcted segments in segmental circumferential strain calculations (right column of Fig. 1a, b) as well as in the corresponding short axis cine images, recognizing visual wall motion abnormalities (VWMA) as previously described [3]. Datasets of all patients (AMI and FU exams) and controls were mixed and presented in random order to both readers. Both readers were blinded to each other, to LGE/edema images (Fig. 1a, b, left column) and to clinical information.

#### Assessment of affected segments in LGE images and T2w

In a separate session, both readers had to define affected segments (short axis LGE, black-blood T2-weighted images with fat saturation), including classification of affected segments in LGE images in viable (below 50% infarcted wall thickness) or non-viable segments (more than 50% infarcted wall). Readers were blinded to clinical information and each other. Reference standard was the existing corresponding CMR report, revised by a cardiologist with over 15 years of experience in CMR (EACVI level III certified). Ventricular volumes and function were calculated using IntelliSpace Portal, performed by reader A (Philips, Version 8.0.3; Table 1a, b).

### Statistical analyses

Statistics were performed using commercially available software (IBM SPSS Statistics, release 25.0; SPSS, Armonk, NY). Categoric data are expressed as numbers or percentages and quantitative data are expressed as means ± standard deviations. Normal distribution was tested by the Kolmogorov–Smirnov test. Two-tailed paired *t* tests or Wilcoxon signed rank were used to compare global and segmental strain values as well as to compare infarcted segments found in LGE, circumferential strain calculations and by visual wall motion assessment. Interobserver agreement was investigated using the intraclass correlation coefficient (ICC). ICC = 0.50–0.75 was considered moderate, ICC = 0.75–0.9 was considered good and ICC > 0.9 was considered excellent agreement [10]. Receiver operating characteristics (ROC) were calculated to determine the cut-offs of segmental strain values and area under the curve (AUC) for segmental strain (SPCS, SPRS and SPLS) in order to differentiate infarcted from remote myocardium. Statistical significance was supposed at a *p* value below 0.05.

## Results

### LGE and edema

In patients with acute infarction, 189 out of 896 segments showed LGE (21.1%) and myocardial edema. Myocardial edema was also detected in 27 segments without LGE. Mean scar burden per patient was 23.4% ± 6 (range 8–59%), the average amount of infarcted segments per patient was 3.7 (range: 2–9 segments) and most infarcted segments were considered non-viable (186/189) (Table 1a).

In the subgroup of patients with follow-up exams 118 out of 512 segments showed LGE (23%). Mean scar burden at acute imaging timepoint was 25.1% ± 5 per patient (range 12–56%) with mostly non-viable scars (117/118), further 10 segments had myocardial edema without concomitant LGE. Scar burden decreased in follow-up exams (20.7 ± 4, range 5–48%) and 15 segments were reclassified from non-viable in AMI exams to viable in FU (Table 1b). No LGE was found in the control group.

### Global strain

**Fig. 2:**
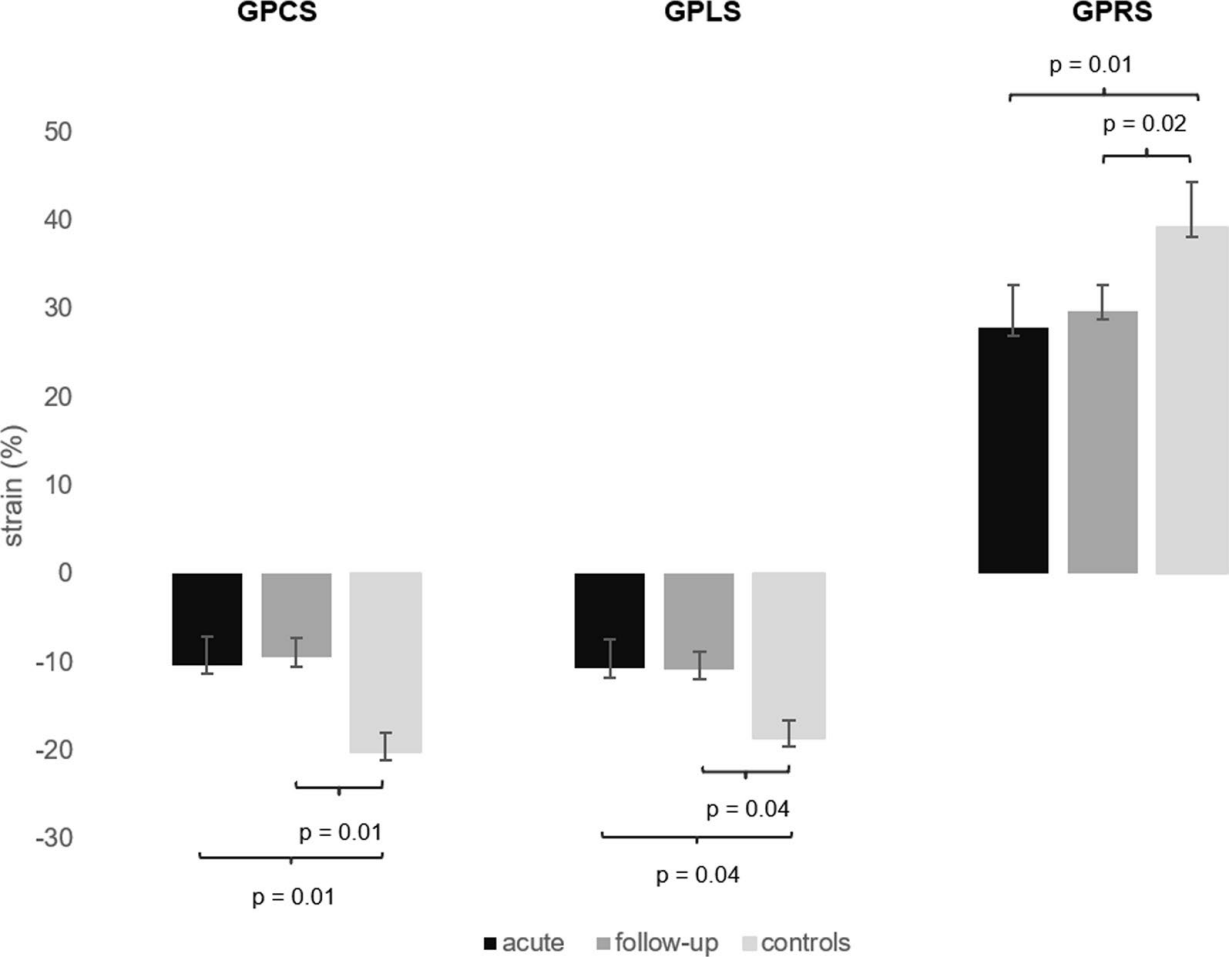
Global strain values in patients and healthy controls. While GPCS, GPLS and GPRS values were very similar comparing both imaging time points, they were significantly impaired compared to healthy controls. *GPCS* global peak circumferential strain, *GPLS* global peak longitudinal strain, *GPRS* global peak radial strain

In patients, mean global strain was impaired compared to controls (global peak circumferential strain [GPCS]: − 10.3% ± 3 vs. − 19.9% ± 2, *p* = 0.01; global peak longitudinal strain [GPLS]: − 10.7% ± 5 vs. − 18.9% ± 4, *p* = 0.04; global peak radial strain [GPRS]: 27.9% ± 5 vs. 39.8% ± 6; *p* = 0.01, Fig. 2). In the subgroup with follow-up CMR, similar mean global strain values were measured between both time points (GPCS: − 10.6% ± 2 vs. − 9.5% ± 3, *p* = 0.7; GPLS − 10.2% ± 5 vs. − 10.9% ± 5, *p* = 0.8; GPRS 26.8% ± 6 vs. 29.8% ± 4; *p* = 0.2; Fig. 2).

### Segmental strain

#### Segmental strain in patients with AMI

**Fig. 3:**
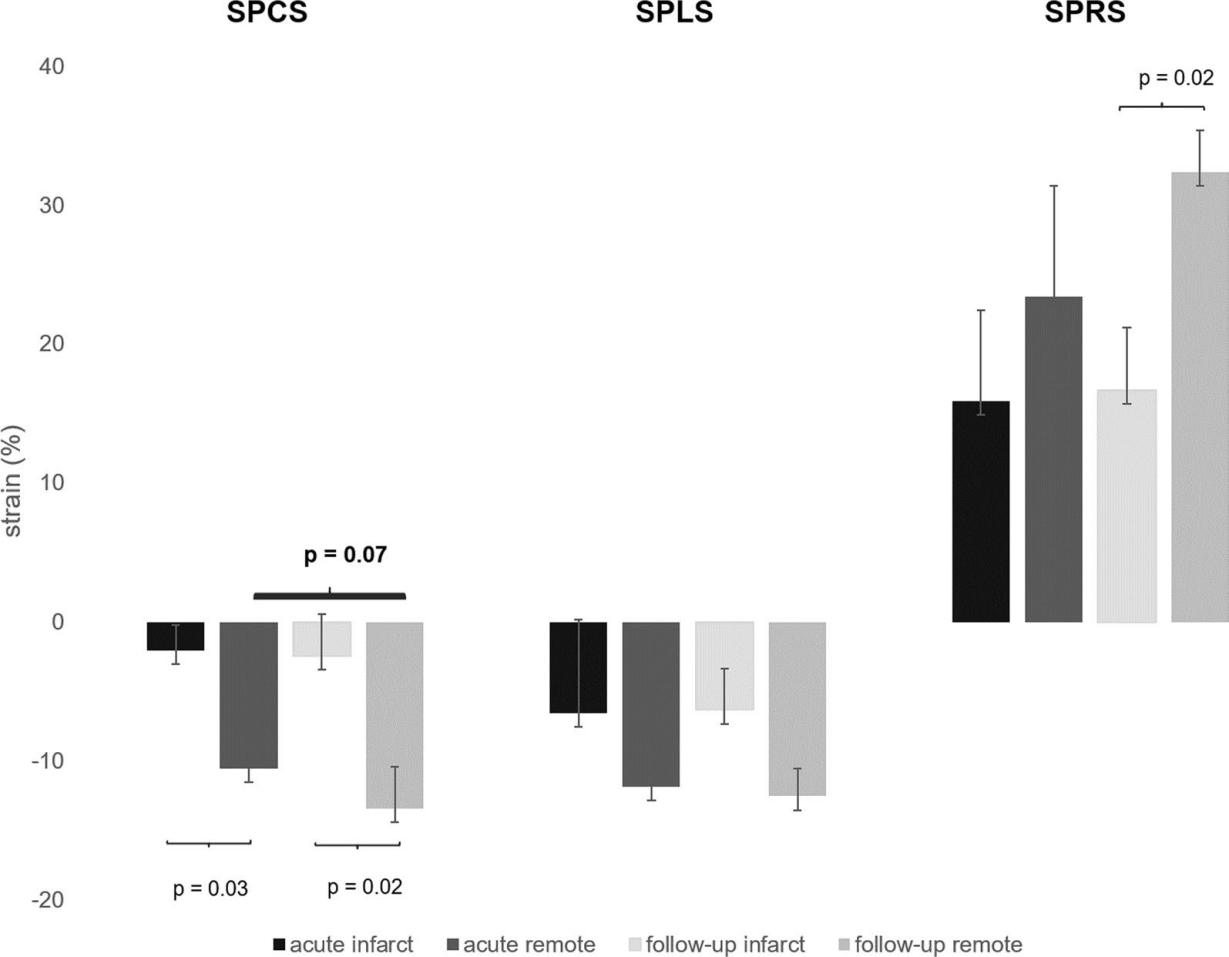
Segmental strain values for infarcted segments and remote myocardium in acute and follow-up CMR. Significantly different values between infarcted and remote myocardium can be detected in SPCS for both imaging time points as well as in SPRS in the follow-up exams. *SPCS* segmental peak circumferential strain, *SPLS* segmental peak longitudinal strain, *SPRS* segmental peak radial strain

**Fig. 4:**
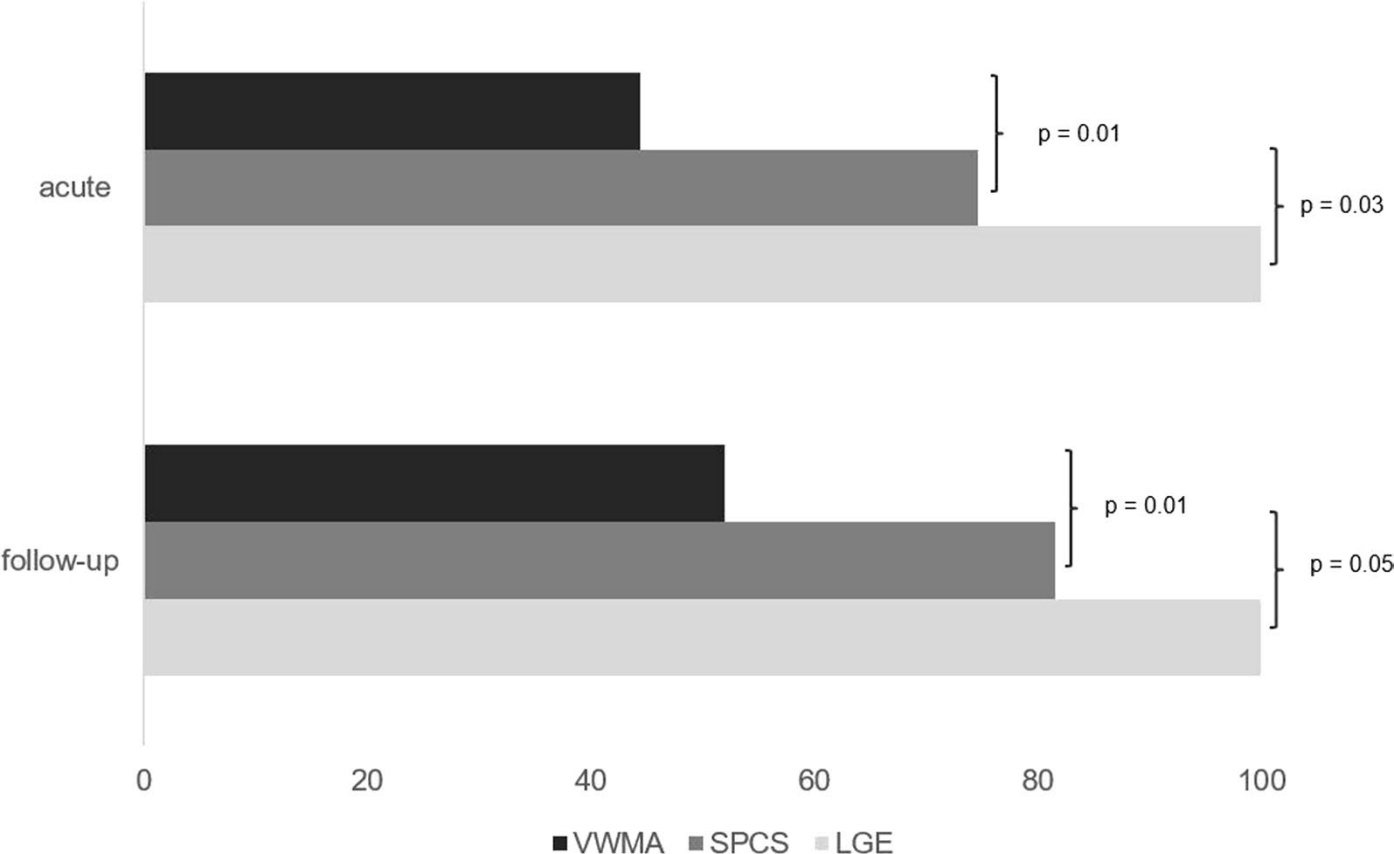
Localization of infarcted segments showed in segmental circumferential strain calculations. Segmental strain calculations showed significantly more infarcted segments than visual assessment of wall motion abnormalities in cine images and this was significant in both imaging time points. In follow-up exams more infarcted segments were found in visual assessment of wall motion compared to acute infarcts (52% vs. 44.4%). *LGE* late gadolinium enhancement, *SPCS* segmental peak circumferential strain, *VWMA* visual wall motion assessment

In patients with AMI, mean segmental peak circumferential strain (SPCS) was markedly impaired in infarcted segments compared to mean SPCS of healthy myocardium (− 2% ± 1 vs. − 10.5% ± 1, *p* = 0.03, Fig. 3), interobserver agreement was excellent (Table 2)*.* Mean segmental peak longitudinal strain (SPLS) and mean segmental peak radial strain (SPRS) in infarcted segments were mildly impaired (SPLS—6.5% ± 8 and SPRS 15.9% ± 7) compared to SPLS and SPRS of remote myocardium (SPLS − 11.8% ± 5 and SPRS 23.4% ± 7, *p* = 0.7 and 0.5) (Fig. 3). From 189 segments with LGE, 141 could be identified in cine based segmental circumferential strain calculations (74.6%; ICC 0.869, 95% CI 0.811–0.908). Moreover, both readers detected all patients with scars in strain calculations, the “missed” 48 segments belonged to patients, that were already diagnosed with at least one infarcted segment. 15 segments were assumed “infarcted” in circumferential strain calculations without displaying LGE, all those segments had myocardial edema. Visual assessment of wall motion abnormalities (VWMA) in cine images revealed 82 infarcted segments out of 189 (43.4%; ICC 0.789, 95% CI 0.729–0.821; Fig. 4). No normal segments (without edema and LGE) in patients nor segments in controls were assumed infarcted by VWMA or circumferential strain calculations.

**Table 2:** Interobserver agreement

	ICC acute	ICC follow up
Global strain		
GPCS	0.902 [95% CI 0.878–0.930]	0.916 [95% CI 0.882–0.941]
GPLS	0.850 [95% CI 0.817–0.879]	0.878 [95% CI 0.804–0.929]
GPRS	0.893 [95% CI 0.851–0.939]	0.897 [95% CI 0.878–0.947]
Segmental strain		
SPCS	0.899 [95% CI 0.862–0.922]	0.903 [95% CI 0.869–0.934]
SPLS	0.732 [95% CI 0.711–0.749]	0.719 [95% CI 0.701–0.747]
SPRS	0.804 [95% CI 0.793–0.869]	0.817 [95% CI 0.797–0.902]

*GPCS/GPLS/GPRS* global circumferential/longitudinal/radial strain, *SPCS/SPLS/SPRS* segmental circumferential/longitudinal/radial strain, *ICC* intraclass correlation coefficient

#### Segmental strain in follow-up CMR

In FU exams, mean SPCS and SPRS were also significantly impaired in infarcted segments compared to SPCS and SPRS of remote myocardium (SPCS − 2.4% ± 2 vs. − 13.4% ± 2, *p* = 0.02; SPRS 16.7% ± 4 vs. 32.4% ± 3, *p* = 0.02; Fig. 3) with excellent interobserver agreement (Table 2). Viable scars had only subtle SPCS impairment (SPCSviable − 8.1% ± 4 vs. SPCS − 2.4% ± 2, *p* = 0.1). Direct comparison between imaging in the acute setting and in follow-up CMR revealed no significant differences in segmental strain values between infarcted segments and remote myocardium, however, a tendency towards lower segmental circumferential strain of remote myocardium in the acute subgroup was noticeable (AMI − 10.6% ± 1 vs. FU exam − 12.9% ± 2, *p* = 0.07; Fig. 3).

**Fig. 5:**
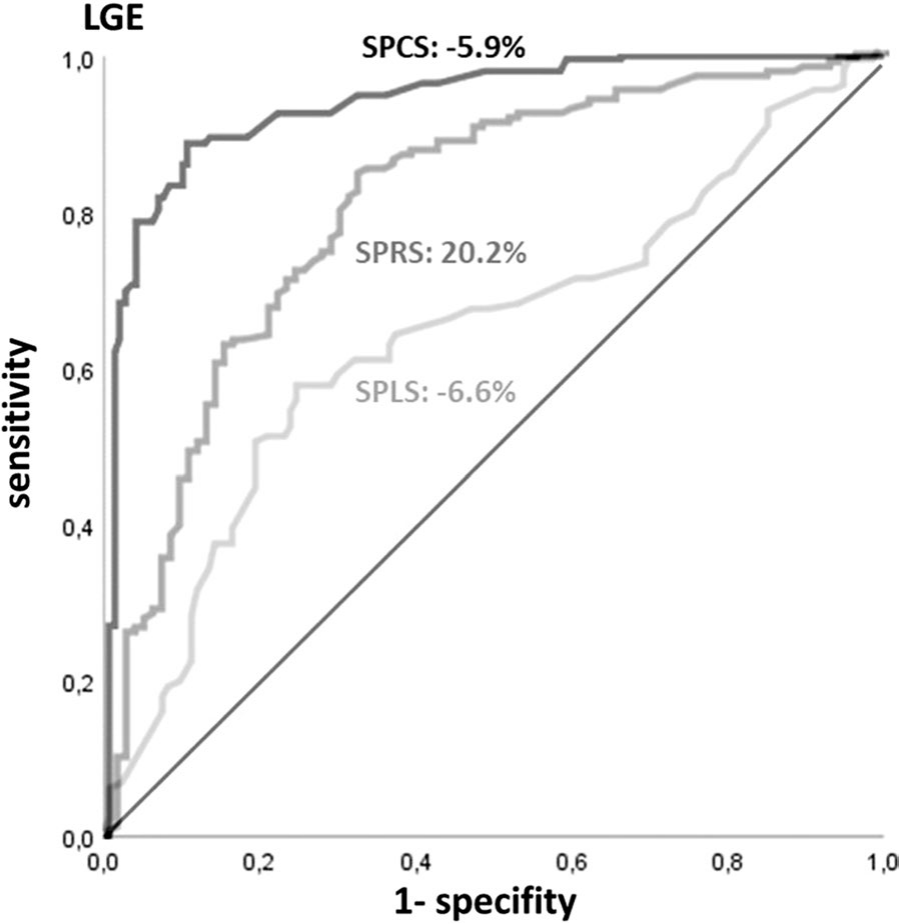
ROC curves for distinguishing infarcted and remote myocardium based on segmental strain parameters. Below a SPCS value of − 5.9% (sensitivity of 86.2%, specificity of 83.5%, AUC 0.89 [95% CI 0.878–0.923, *p* < 0.05]) segments are considered infarcted. The cut-off value for SPRS was 20.2% (sensitivity of 77.5%, specificity of 77.9%, AUC 0.78 [95% CI 0.718–0.814, *p* < 0.05]) and − 6.6% for SPLS (sensitivity of 60.4%, specificity of 72.9%, AUC 0.66 [95% CI 950.598–0.715, *p* < 0.05]). Infarcted segments in LGE were considered gold standard. *LGE* late gadolinium enhancement, *ROC* receiver operating characteristic, *SPCS/SPRS/SPLS* segmental peak circumferential/radial/longitudinal strain

ROC analyses were performed for SPCS, SPRS and SPLS to detect the optimal cut-off values for discrimination of infarcted segments and remote myocardium; infarcts visible in LGE were considered reference standard (Fig. 5). A SPCS value below − 5.9% was considered infarcted (sensitivity of 86.2%, specificity of 83.5%, AUC 0.89 [95% CI 0.878–0.923, *p* < 0.05]). The cut-off value for SPRS was 20.2% (sensitivity of 77.5%, specificity of 77.9%, AUC 0.78 [95% CI 0.718–0.814, *p* < 0.05]) and for SPLS − 6.6% (sensitivity of 60.4%, specificity of 72.9%, AUC 0.66 [95% CI 0.598–0.715, *p* < 0.05]).

Evaluation of segmental circumferential strain calculations detected 91 out of 114 infarcted segments (80%; ICC 0.865, 95% CI 0.802–0.908), detection of VWMA in cine sequences revealed 59 segments (51.8%; ICC 0.802, 95% CI 0.759–0.831; Fig. 4). Both readers missed one patient with subtle viable scar (one infarcted segment) in segmental circumferential strain calculation.

## Discussion

This study analyzed the feasibility of using segmental strain for scar detection in patients with acute and subacute myocardial infarcts. Segmental circumferential strain calculations based on native cine images detected all patients with AMI and 80% of infarcted segments in subacute follow-up exams.

In the clinical setting, established alternatives for scar detection in native CMR sequences are limited. With native T1 mapping, scar and remote myocardium can be differentiated due to different tissue relaxation times [11, 12]. However, additional mapping sequences need to be acquired and in order to achieve accurate measurements standardized parameters for healthy myocardium need to be defined separately for every scanner. Moreover, while acute infarcts can be reliably detected in native T1 maps, T1 values of infarcted areas normalize after acute infarction with resulting lower specificity for chronic infarcts [13]. Some artificial intelligence-based techniques successfully detected scar tissue in non-contrast cine CMR sequences [14, 15], but these methods are mostly still in a proof-of-concept stage and are not yet practicable in clinical use.

Myocardial feature tracking (FT) was introduced as a novel technique for myocardial strain quantification based on routinely acquired cine sequences. Infarcted tissue leads to altered global and segmental myocardial strain due to reduced contractility of fibroblasts, that gradually replace necrotic myocardium after myocardial infarction [6]. Impairment of global strain in patients with acute and chronic infarcts has been reported by various studies [16, 17]. Accordingly, GPLS, GPRS and especially GPCS was impeded in our patient cohort compared to healthy controls. Studies analyzing segmental strain in patients with infarcts in the last decade revealed heterogenous results, in particular problems with accuracy and reproducibility of segmental strain values have been reported [18]. Newer algorithms for strain quantification based on non-rigid algorithm for image registration and segmentation with tracking of the whole image content—instead of tracking myocardial borders only-seem to accurately identify scarred myocardium in segmental circumferential strain [19, 20].

Chronic scars with wall motion abnormalities and myocardial wall thinning lead to severe impairment of regional deformation parameters in contrast to healthy tissue, allowing distinction of remote and infarcted segments in regional strain measurements [2, 7].

However, the impact of acute infarcts on segmental strain in native cine images has not yet been sufficiently investigated. In contrast to chronic infarcts, which may be visible with the bare eye in cine images due to wall thinning or dyskinesia and are characterized by replacement fibrosis, acutely infarcted myocardium with its various pathophysiologic processes including necrosis and edema mostly lacks wall thinning and has often only subtle wall motion abnormality [21, 22]. Therefore, possible strain impairment in acute infarcts is apparently based on different mechanisms compared to strain impairment in chronic scars. Nevertheless, similar to chronic infarcts, regional mechanical impairment of acutely infarcted myocardium was best reflected in circumferential strain calculations [23]. In our patient cohort, mean SPCS in infarcted tissue was significantly impaired compared to SPCS of remote myocardium and this was observed in both acute imaging as well as in subacute follow-up CMRs. Comparing both exams, infarcted tissue showed similar mean SPCS values, remote myocardium on the other hand showed slightly more impairment in the acute imaging timepoint. Further analyses revealed that edematous segments adjacent to infarcts caused strain impairment, suggesting influence of myocardial edema on segmental circumferential strain. Accordingly, false positive classification of edematous segments as “infarcted” by both readers was observed in the acute timepoint. After edema subsided in follow-up exams, no false positive results were noticed.

Similar to chronic infarcts, infarcted segments could be distinguished from healthy myocardium with high sensitivity and specificity below a calculated threshold in SPCS calculations, while sensitivity and specificity was markedly lower for corresponding thresholds in SPRS and SPLS.

Direct comparison of wall motion and segmental circumferential strain calculations of every patient in a blinded dataset revealed markedly more infarcted segments in SPCS calculations than by analyzing cine images only and this was true for the acute timepoint (74.6% vs. 43.4%) as well as in follow-up exams (80% vs. 52%). The higher amount of infarcted segments detected by VWMA in follow-up exams could be explained with the incremental myocardial thinning weeks after infarction.

Perfect sensitivity for the detection of patients with AMI was observed in SPCS calculations, where missed infarcted segments belonged to patients already classified as “patient with infarction” by the readers. In follow-up exams, when LGE burden subsided, some scarred segments were reclassified from non-viable to viable scars. Viable scars showed lesser SPCS impairment than non-viable infarcts and in fact one patient with a small viable scar was classified as “patient with no infarction” in SPCS calculations by both readers. SPCS impairment mainly correlates with damage of circumferentially orientated myocardial fibers, that lay below the superficial subendocardial fibers of the LV myocardium [24, 25]. In viable scars, deeper lying circumferential fibers are probably not enough affected to cause significant SPCS impairment. This is a relevant limitation of this study, since most infarcts in our patient cohort were non-viable. Furthermore, regional deformation parameters detected by segmental strain are influenced by various factors and are not specific for ischemic tissue damage. Further studies are needed to analyse segmental strain in patients with infarcts and concomitant cardiac diseases that are known to influence global strain like cardiomyopathies or storage disease [26, 27]. Moreover, temporary cardiac conditions like myocardial hibernation or stunning or even benign anatomical variants like a left ventricular diverticulum with potential impact on segmental strain needs to be examined, preferably in a prospective setting with a larger patient cohort. In this retrospective study with initially 57 patients, follow-up exams were available in only 32 individuals. The mean interval of 5 weeks between initial imaging and follow-up CMR is presumably not long enough to measure remodelling, because of still ongoing pathophysiologic processes and distant time points should be investigated for that matter in further studies. In addition, segmental circumferential strain calculations use the 16-segment model and apical infarction (segment 17) cannot be detected in SPCS calculations.

Ultimately, strain measurements were performed with only one software. Recent studies show, that strain values are not interchangeable between different vendors, thus vendor-specific threshold values need to be defined for infarcted and remote myocardium [20].

## Conclusion

Segmental circumferential strain derived from routinely acquired non-contrast cine sequences detects nearly 75% of acute infarcts and 80% of infarcts in subacute follow-up CMR, significantly more than visual evaluation of cine images alone. Especially in acute infarcts, where wall motion abnormalities may be subtle and wall thinning is not yet present, this technique may aid infarct detection in patients with ischemic heart disease, who cannot receive or reject gadolinium application or when LGE images are non-diagnostic. However, since strain impairment is not specific for ischemic tissue damage, further studies are needed to investigate if this technique can be used in patients with concomitant cardiac conditions (e.g. cardiomyopathies) where global strain parameters may be altered.

## Data Availability

The datasets generated and analysed during this study are not publicly available due to their patient referable character, thereby compromising individual privacy, but are available from the corresponding author on reasonable request.

## Abbreviations

AMI: Acute myocardial infarction
AUC: Area under the curve
CMR: Cardiac magnetic resonance
FT: Feature tracking
FU: Follow-up (exam)
GPCS: Global peak circumferential strain
GPLS: Global peak longitudinal strain
GPRS: Global peak radial strain
ICC: Intraclass correlation coefficient
i.v.: Intravenous
LGE: Late gadolinium enhancement
LV: Left ventricle/left-ventricular
LVEDV: Left ventricular end-diastolic volume
LVEF: Left ventricular ejection fraction
LVESV: Left ventricular end-systolic volume
LVSV: Left ventricular stroke volume
ms: Milliseconds
min: Minute(s)
ROC: Receiver operating characteristics
s: Seconds
SPCS: Segmental peak circumferential strain
SPLS: Segmental peak longitudinal strain
SPRS: Segmental peak radial strain
SSFP: Steady-state free precession
T: Tesla
VWMA: Visual wall motion abnormality
